# Embarrassingly Parallel Acceleration of Global Tractography via Dynamic Domain Partitioning

**DOI:** 10.3389/fninf.2016.00025

**Published:** 2016-07-13

**Authors:** Haiyong Wu, Geng Chen, Yan Jin, Dinggang Shen, Pew-Thian Yap

**Affiliations:** ^1^School of Information Engineering, Xiaozhuang University, Nanjing, China; ^2^Department of Radiology and Biomedical Research Imaging Center (BRIC), University of North Carolina, Chapel Hill, NC, USA; ^3^Department of Brain and Cognitive Engineering, Korea University, Seoul, South Korea

**Keywords:** diffusion magnetic resonance imaging, global tractography, Markov chain Monte Carlo, brain connectivity, parallel computing

## Abstract

Global tractography estimates brain connectivity by organizing signal-generating fiber segments in an optimal configuration that best describes the measured diffusion-weighted data, promising better stability than local greedy methods with respect to imaging noise. However, global tractography is computationally very demanding and requires computation times that are often prohibitive for clinical applications. We present here a reformulation of the global tractography algorithm for fast parallel implementation amendable to acceleration using multi-core CPUs and general-purpose GPUs. Our method is motivated by the key observation that each fiber segment is affected by a limited spatial neighborhood. In other words, a fiber segment is influenced only by the fiber segments that are (or can potentially be) connected to its two ends and also by the diffusion-weighted signal in its proximity. This observation makes it possible to parallelize the Markov chain Monte Carlo (MCMC) algorithm used in the global tractography algorithm so that concurrent updating of independent fiber segments can be carried out. Experiments show that the proposed algorithm can significantly speed up global tractography, while at the same time maintain or even improve tractography performance.

## 1 Introduction

Diffusion magnetic resonance imaging (DMRI) (Basser et al., 1994) relies on the fundamental observation that the diffusion of water molecules in white matter (WM) is much less restricted along the direction of axonal bundles than perpendicular to them. DMRI is widely used as a non-invasive imaging modality for studying WM changes in association with development, growth, and disorders, such as Alzheimer’s disease (Li et al., 2013; Termenon et al., 2013; Jin et al., 2015a,b), Parkinson’s disease (Martínez-Murcia et al., 2014), multiple sclerosis (Goldberg-Zimring et al., 2005), autism (Thomas et al., 2011), and traumatic brain injury (Dennis et al., 2015a,b). DMRI is also used to map out the comprehensive wiring of the brain, generating what is commonly known as the brain connectome (Van Essen et al., 2013; Nossenson et al., 2016).

Diffusion tractography algorithms estimate the WM pathways by constructing streamlines that trace through the directional information at each voxel given by DMRI data (Conturo et al., 1999; Mori et al., 1999; Basser et al., 2000). They can be generally divided into two categories: local tractography (LT) and global tractography (GT). LT starts from a random or predetermined region and traces local voxel-wise fiber orientations in small successive steps. At each voxel, there can be a single direction, e.g., based on the principal direction of the tensor model (Mori et al., 1999; Basser et al., 2000), or multiple directions as given by the peaks of the orientation distribution function (ODF) (Tuch, 2004). The tracing of these directions can be done deterministically (Mori et al., 1999; Basser et al., 2000) or probabilistically (Behrens et al., 2003; Parker et al., 2003). The main strength of LT lies in its speed. For example, whole-brain LT takes only a few minutes with the FACT algorithm (Mori et al., 1999). However, LT is susceptible to error accumulation owing to the estimation uncertainty of the directions at each voxel (Behrens et al., 2003). This causes the estimated tracts to deviate from the true WM trajectories.

On the other hand, GT (Reisert et al., 2009, 2011) uses global optimization techniques to decrease sensitivity to local estimation errors. GT reconstructs all fiber trajectories simultaneously by considering their agreement with the underlying diffusion data. The reconstructed fiber trajectories are the result of the interaction between signal-generating fiber segments and their matching with the diffusion-weighted measurements. The inter-segment interaction is reflected by the *internal energy* and the matching of the signals of the fibers with the data is reflected by the *external energy*. The configuration of these fiber segments is optimized globally by minimizing the global energy, computed as the sum of the internal energy and the external energy (Kreher et al., 2008; Fillard et al., 2009; Reisert et al., 2009, 2011). GT algorithms can better resolve ambiguous WM pathways and afford better robustness to imaging noise (Mangin et al., 2013). Utilizing a Monte Carlo optimization framework, GT randomly perturbs the fiber segments using a creation/deletion, connection/disconnection, and shifting mechanism to determine a configuration of the fiber segments that best fits the data. Although studies have shown that GT generally outperforms LT (Fillard et al., 2011), the main drawback of GT methods is their computational costs. Whole-brain GT takes from a few hours up to a day on a standard machine (Reisert et al., 2011; Neher et al., 2012). Therefore, reducing the computational cost of GT is critical to improving its usability in clinical settings.

The key idea of recent works on accelerating LT algorithms is to generate fiber trajectories from the seed voxels in parallel instead of sequentially (Lee and Kim, 2013). However, this approach cannot be applied to GT because of the interdependence of the fiber tracts in the global optimization framework. The GT algorithm has been recently modified in different forms with multithreading capability (Reisert et al., 2014; Christiaens et al., 2015). It is however unsure whether in these implementations the statistical independence structure of the problem has been taken into account to allow a mathematically valid parallelization of the associated MCMC algorithm.

In this work, we will focus on accelerating the GT algorithm proposed by Reisert et al. (2011). This algorithm utilizes a Markov chain Monte Carlo (MCMC) technique, called the Metropolis–Hastings algorithm (Neal, 1993; Van Lieshout, 2000), to determine the configuration of fiber segments with the minimum global energy. In theory, the Markov process asymptotically reaches a unique stationary distribution that equals the posterior distribution of the fiber configuration given the data. However, MCMC methods can be prohibitively slow and require a large number of “burn-in” steps before producing representative samples.

An *embarrassingly parallel* approach was recently proposed to parallelize burn-in and sampling in MCMC (Neiswanger et al., 2014). The key idea is to apply any classical MCMC method independently to subsets of data without requiring much communication between them. First, the data are partitioned into multiple subsets. Next, an MCMC method is used to draw samples from *a posteriori* distribution given by each subset. Finally, the samples from all of the subsets are combined to form samples from the full posterior. This method is termed *embarrassingly parallel* because the processing of each subset is performed independently without communication with other subsets until the final combination stage.

Building on this concept, we show in this work that the GT algorithm can be improved significantly in terms of speed by MCMC parallelization. The key observation that drives our algorithm, called the parallel global tractography algorithm (PGT), is that the spatial extent of the influence of each fiber segment is limited. That is, the influence of each fiber segment depends only on the fiber segments that are connected (or can potentially be connected) to its both ends and also on the diffusion-weighted signal that is in its proximity. In other words, despite the fact that we try to decrease the total fitting energy in a global sense, the influence of each fiber segment on the variation of the energy is in fact *local*. Based on this observation, significant parallelism can be harnessed for improving the speed of the GT algorithm. The data can be partitioned into subsets similar to Neiswanger et al. (2014) and processed separately before combining the results to form samples for the original problem. Experimental results confirm the effectiveness of the proposed method and demonstrate that comparable tractography performance can be achieved in a reduced amount of time.

Part of this work has been reported in our recent workshop paper (Wu et al., 2015). Herein, we provide additional examples, results, derivations, and insights that are not part of the workshop publication.

## 2 Accelerating Global Tractography

### 2.1 Background

**Figure 1:**
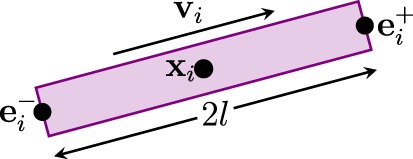
**A fiber segment**.

The GT algorithm assumes that a fiber streamline is composed of fiber *segments*, each of which can be represented by a cylinder, as illustrated in Figure 1. The *i*-th segment can be defined as a tuple *h_i_* = (**x***_i_*, **v***_i_*, *l*, *d*), where **x***_i_* ∈ R^3^ denotes the spatial location of the center of the segment, **v***_i_* ∈ *S*_2_ denotes the orientation, *l* is the half length, and *d* is the diameter. Both the length and the diameter are identical for all segments. The extremities of the fiber segment are ${\text{e}}_{i}^{\alpha }$, where *α* ∈ {+, −} indicate the positive or negative endpoint.

The goal of GT (Reisert et al., 2011) is to determine the optimal configuration M of a set of signal-generating fiber segments given the measured diffusion-weighted signals D, including their existence, spatial positions, orientations, and connections at both ends to other fiber segments. More formally, one is interested in determining the M that maximizes the posterior probability *P*(M|D) defined as

(1) $$P\left(M|D\right)\;\propto \;P\left(M\right)P\left(D|M\right)\;=\;\exp\left(\;\frac{-E_{\text{int}}\left(M\right)\;-\;E_{\text{ext}}\left(M,D\right)}{T}\;\right)\;,$$

where *E*_int_(M) and *E*_ext_(M, D) are the internal energy and the external energy, respectively, and *T* is the temperature associated with simulated annealing (Aarts and Korst, 1989).

The internal energy *E*_int_(M) characterizes the smoothness of the fibers and is defined as the sum of all the interaction potentials between connected segments:

(2) $$E_{\text{int}}\left(M\right)=\lambda_{\text{int}}\underset{\left({\text{e}}_{i}^{\alpha_{i\to j}},{\text{e}}_{j}^{\alpha_{j\to i}}\right)}{\sum }\;\frac{1}{l^{2}}\left(||{\text{e}}_{i}^{\alpha_{i\to j}}-{\bar{\text{x}}}_{\mathrm{ij}}|{|}^{2}+||{\text{e}}_{j}^{\alpha_{j\to i}}-{\bar{\text{x}}}_{\mathrm{ij}}|{|}^{2}\right)-L,$$

where ${\bar{\text{x}}}_{\mathrm{ij}}$ represents the midpoint of the line that connects the centers of these two segments, and ${\text{e}}_{i}^{\alpha_{i\to j}}$ denotes that the endpoint of the *i*-th segment that is connected to the *j*-th fiber segment. The bias *L* affects the probability of connections between segments.

The signal predicted by the fiber segments at location **x** and orientation **v** is defined as

(3) $$F_{M}\left(\text{x},\text{v}\right)=w\sum_{i=1}^{N}\exp\left(-c{\left({\text{v}}^{\text{T}}{\text{v}}_{i}\right)}^{2}\right)\exp\left(\frac{-|\text{x}-{\text{x}}_{i}{|}^{2}}{\sigma^{2}}\right).$$

where *N* is the total number of fiber segments. The constant *w* controls the amount of signal contribution from each fiber segment. Parameter *σ* > 0 controls the spatial extent of the influence of each fiber segment. Parameter *c* > 0 controls the shape of the signal profile generated by each fiber segment (Reisert et al., 2011). In practical implementation, the second exponential term is truncated and hence the spatial extent is limited.

The external energy *E*_ext_(M, D) measures the difference between the observed data D and the predicted signal *F*_M_ (**x**, **v**) given by the configuration M:

(4) $$E_{\text{ext}}\left(M,D\right)=\lambda_{\text{ext}}||F_{M}-D|{|}^{2}=\lambda_{\text{ext}}\int_{R^{3}\times S_{2}}\;|F_{M}\left(\text{x},\text{v}\right)-D\left(\text{x},\text{v}\right){|}^{2}d^{3}\text{x}d^{2}\text{v},$$

where λ_ext_ is a tuning parameter.

### 2.2 Parallel Global Tractography

To maximize the posterior probability *P*(M|D), an MCMC method called the Metropolis–Hastings (MH) algorithm (Metropolis et al., 1953; Hastings, 1970) is employed in Reisert et al. (2011). However, MCMC methods may take a prohibitively long time, depending on the number of data points. Furthermore, MCMC methods might require a large number of “burn-in” steps before beginning to generate representative samples (Liu, 2001). Here, we propose a parallelized version of the GT algorithm, called parallel global tractography (PGT), to improve the speed.

PGT utilizes the structure of the problem to allow embarrassingly parallel speed up of the GT algorithm. Similar to Neiswanger et al. (2014), this is achieved by partitioning the data into subsets, on which an MCMC algorithm can act independently without communication between them until the final combination stage. More formally, this is done by partitioning the data D into *K* subsets {D_1_, D_2_, … , D*_K_*} and associate with each subset a *subposterior* probability $P{\left(M\right)}^{\frac{1}{K}}P\left(D_{k}|M\right)$. MCMC sampling is then performed independently for each *subposterior* probability before eventually combining their samples to produce samples from an estimate of the subposterior density product, which is proportional to the full-data posterior. If the density product estimator is consistent, it can then be shown that one is drawing asymptotically exact samples from the full posterior distribution. Parametric, non-parametric, and semiparametric estimation techniques are described in Neiswanger et al. (2014).

While straightforward, the method described above (Neiswanger et al., 2014) is not directly applicable to the parallelization of GT. The problem lies in the difficulty in designing an appropriate consistent estimator of the subposterior density estimator that will allow us to draw asymptotically exact samples from the posterior. Combination of the samples drawn from the subposteriors is further complicated by the fact that the dimensionality of the parameters of the configuration M is not fixed due to the creation/deletion proposals (Kreher et al., 2008). In fact, for this reason, GT requires a reversible jump version of MCMC (Green, 1995). In PGT, we overcome this problem by leveraging the structure of the GT problem to further improve parallelism. We first rewrite the posterior probability as

(5) $$P\left(M|D\right)=P\left(M_{0},M_{1},...,M_{K}|D\right)$$

(6) $$=P\left(M_{1},...,M_{K}|D,M_{0}\right)P\left(M_{0}|D\right).$$

**Figure 2:**
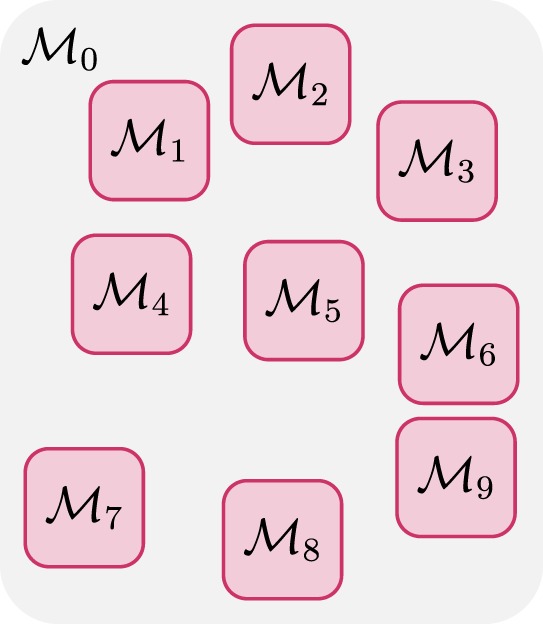
**Domain partitioning (*K* = 9)**.

Note that here M_0_ denotes the configuration of the fiber segments between the other *K* regions, in which the fiber configurations are denoted as {M_1_, M_2_, … , M*_K_*} (see Figure 2). If the region covered by M_0_ gives sufficient separation between the *K* regions, and noting that each fiber segment is affected by its neighboring and not distant fiber segments (as required by the internal energy), we have

(7) $$P\left(M|D\right)=P\left(M_{0}|D\right)\prod_{k=1}^{K}P\left(M_{k}|D,M_{0}\right).$$

This indicates that the configurations {M_1_, … , M*_K_*} are conditionally independent given D and M_0_. Noting the fact that the configuration of each fiber segment is only dependent on the data in its vicinity, we further have

(8) $$P\left(M|D\right)=P\left(M_{0}|D_{0}\right)\prod_{k=1}^{K}P\left(M_{k}|D_{k},M_{0}\right).$$

This implies that once the samples for M_0_ have been drawn, the samples for {M_1_, M_2_, … , M*_K_*} can be drawn independently and hence in parallel. In contrast to Neiswanger et al. (2014), instead of partitioning the data, we partition the parameters according to the spatial location and then partition the data accordingly. This formulation allows us to make local changes (creation/deletion, connection/disconnection, and shifting) in parallel. We can switch to *K* = 0 to accommodate for more global proposals like the connection/disconnection proposal (Reisert et al., 2009).

The MH algorithm is applied in parallel by proposing changes to the fiber segments in the subregions associated with {M_1_, M_2_, … , M*_K_*}. The proposals in these subregions are accepted or rejected based on their individual acceptance ratios. The independence condition guarantees that the proposals for each of the *K* subregions can be accepted and rejected separately but in parallel.

Then, for *k* = 1, … , *K*, samples are drawn from the subposterior densities *P*(M*_k_*|D*_k_*, M_0_). With D*_k_* and M_0_ fixed and hence *P*(D*_k_*, M_0_) being a constant, we have

(9) $$P\left(M_{k}|D_{k},M_{0}\right)\propto P\left(M_{k},M_{0}\right)P\left(D_{k}|M_{k},M_{0}\right).$$

Proposals for modification of configuration are made for the fiber segments in each region according to its subposterior density by randomly selecting at each time a fiber segment, perturbing it using a creation/deletion, connection/disconnection, and shifting mechanism, and examining if the regional energy can be decreased. In this process, M_0_ remains fixed and {M*_k_*} are updated. After {M*_k_*} are sufficiently updated, they are combined to form M. The random partitionining of the image space into subregions is performed iteratively so that each time the fiber configurations of a different set of *K* random subregions can be updated. The decision of whether to accept a proposal is based on the individual Green’s ratio of the *i*-th region

(10) $$R_{k}=\frac{P\left(M_{k}^{\prime }|D_{k},M_{0}\right)Q\left(M_{k}|M_{k}^{\prime }\right)}{P\left(M_{k}|D_{k},M_{0}\right)Q\left(M_{k}^{\prime }|M_{k}\right)},$$

where $Q\left(M_{k}^{\prime }|M_{k}\right)$ is the transition probability associated with the MH algorithm. From Equation (9), the internal energy contributed by the fiber segments in the *k*-th region alone is

(11) $$E_{\text{int}}\left(M_{k}\right)=\lambda_{\text{int}}\underset{\left({\text{e}}_{i}^{\alpha_{i\to j}},{\text{e}}_{j}^{\alpha_{j\to i}}\right)\in N_{k}\times N_{k}}{\sum }\frac{1}{l^{2}}\left(||{\text{e}}_{i}^{\alpha_{i\to j}}-{\bar{\text{x}}}_{\mathrm{ij}}|{|}^{2}+||{\text{e}}_{j}^{\alpha_{j\to i}}-{\bar{\text{x}}}_{\mathrm{ij}}|{|}^{2}\right)-L$$

and the external energy is

(12) $$E_{\text{ext}}\left(M_{k},D_{k}\right)=\lambda_{\text{ext}}\int_{N_{k}\times S_{2}}\;|F_{M_{k}}\left(\text{x},\text{n}\right)-D_{k}\left(\text{x},\text{n}\right){|}^{2}d^{3}\text{x}d^{2}\text{n},$$

where N*_k_* is the region containing all fiber segments associated with M*_k_*.

Note that some proposals are parallelizable and some are not. For each fiber segment, the change in internal energy associated with creation/deletion and shifting proposals is affected only by the fiber segments it is (or will be) connected to. The change in external energy involves only the diffusion signals in a localized neighborhood surrounding the fiber segment. Hence, creation/deletion and shifting proposals can be performed independently and simultaneously in different subregions. However, the connection/disconnection proposals, which attempt to determine new fibers with lower energy, involve a larger spatial extent and are hence more difficult to parallelize. To overcome this problem, we alternate between parallel proposals (i.e., creation/deletion and shifting) and serial proposals (i.e., connection/disconnection) according to MH transition probabilities assigned to them.

### 2.3 Implementation

**Figure 3:**
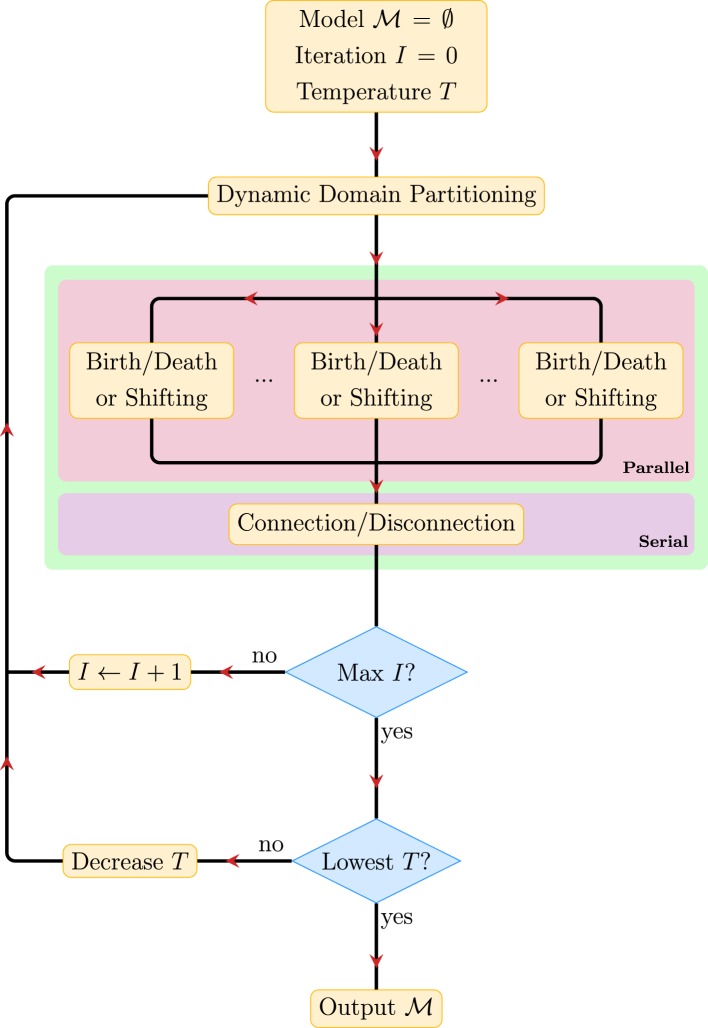
**Overview of PGT**.

Figure 3 summarizes the key steps in PGT. The PGT algorithm involves repeating the following steps until convergence.

1. **Dynamic Domain Partitioning**: Partition the image space into *K* subregions, between which the configurations of the fiber segments are independent.
2. **Parallel Proposals**: Make creation/deletion and shifting proposals in parallel for the fiber segments in these regions according to the corresponding transition probabilities, accept/reject the proposals based on their acceptance ratios, and repeat this step for a sufficient number of times.
3. **Serial Proposals**: Make connection/disconnection proposals and determine fiber tracts that better explain the data.

**Figure 4:**
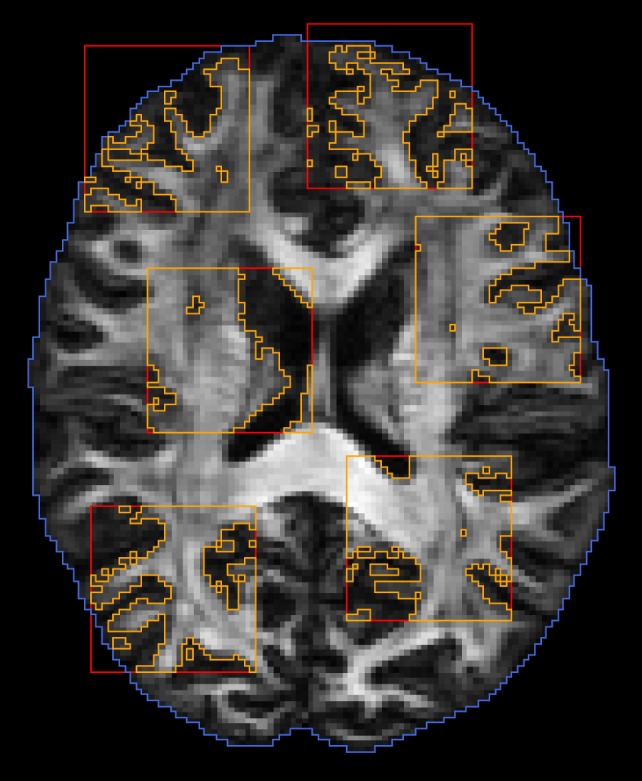
**Dynamic domain partitioning**. Blue: brain region; red: random block regions; orange: regions after applying white matter mask.

We dynamically partition the image data into *K* subregions that are mutually non-influential and statistically independent. We randomly choose *K* points, and the *K* subregions are defined as cubic blocks with these *K* points as centers. These subregions are chosen to be far enough to avoid overlap so as to maintain statistical independence. The region excluding these *K* subregions is the region containing M_0_. After sufficient proposals have been proposed in parallel and in serial, the partitioning is repeated to generate a new set of subregions. Figure 4 shows an example of random subregions with a block size that is typical in our implementation.

## 3 Experiment Results

### 3.1 Datasets

Two synthetic datasets and a real human dataset were utilized to evaluate the performance of PGT.

- *Spiral data*: A 128 × 128 field of diffusion-weighted signals was generated to simulate curved fiber bundles in white matter of human brain, forming a spiral as shown in Figure 8. Each voxel within the spiral was simulated by a tensor model with principal diffusivities λ_1_ = 1.7 × 10^−3^ mm^2^/s, λ_2_ = λ_3_ = 3.0 × 10^−4^ mm^2^/s, and diffusion weighting *b* = 2000 s/mm^2^. The gradient directions and the voxel resolution were identical to those of the real data described below.
- *Cross data*: Another synthetic dataset was generated to simulate two fiber bundles crossing at 90°. The image dimension is 60 × 60, and the signal at each voxel was simulated using a tensor model or its mixture with principal diffusivities λ_1_ = 1.5 × 10^−3^ mm^2^/s, λ_2_ = λ_3_ = 1.0 × 10^−3^ mm^2^/s, and diffusion weighting *b* = 2000 s/mm^2^. The gradient directions and the voxel resolution were the same as those of the real data described below.
- *In vivo data*: Diffusion-weighted images were acquired from an adult with a Siemens 3T Tim Trio MR scanner using an EPI sequence. Diffusion gradients were applied in 120 non-collinear directions with diffusion weighting *b* = 2000 s/mm^2^. The acquisition parameters were: repetition time (TR) = 12,400 ms, echo time (TE) = 116 ms, volume cropped dimensions = 83 × 97 × 76, and voxel resolution = 2 mm × 2 mm × 2 mm.

### 3.2 Results

#### 3.2.1 Iterations Per Partition and Number of Random Partitions

**Figure 5:**
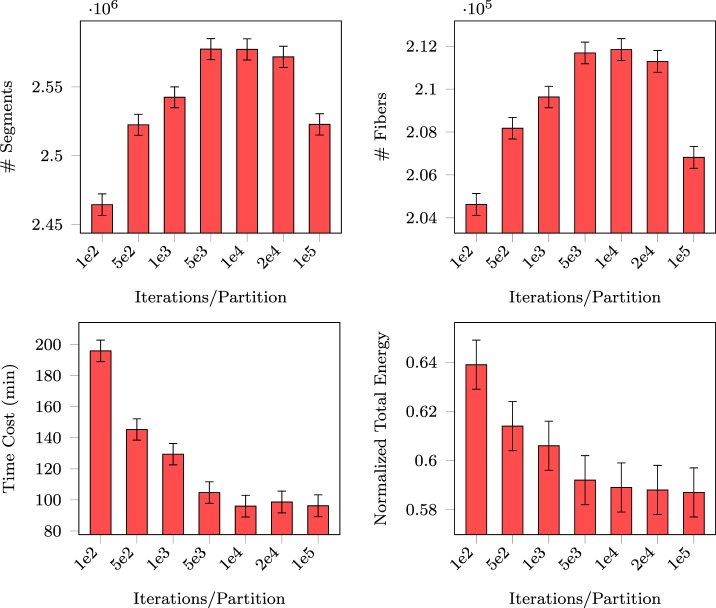
**Number of segments, number of fibers, normalized total energy, and time consumed with respect to different numbers of iterations per partition**.

For fair comparison of PGT with GT, we fixed the total number of proposals that were eventually generated (i.e., 10^8^). For PGT, this means balancing between the number of iterations per partition and the number of random partitions. Since the total number of proposals is proportional to the product of the two quantities, we only need to report the performance of PGT with respect to the former. The number of generated segments, the number of connected fibers, the normalized total energy, and the total computing time are reported in Figure 5. PGT was repeated 10 times using the *in vivo* data with 10 parallel threads.

The figure shows that the numbers of generated segments and connected fibers stabilize at 10^4^ iterations per partition. The figure also indicates that with this amount of per partition iterations the total energy and the time consumed became stable, implying that this is sufficient for MCMC “burn-in.” In the following experiments, we iterated MCMC 10^4^ times for each set of partitioned regions before randomly partitioning the data again.

#### 3.2.2 Energy

**Figure 6:**
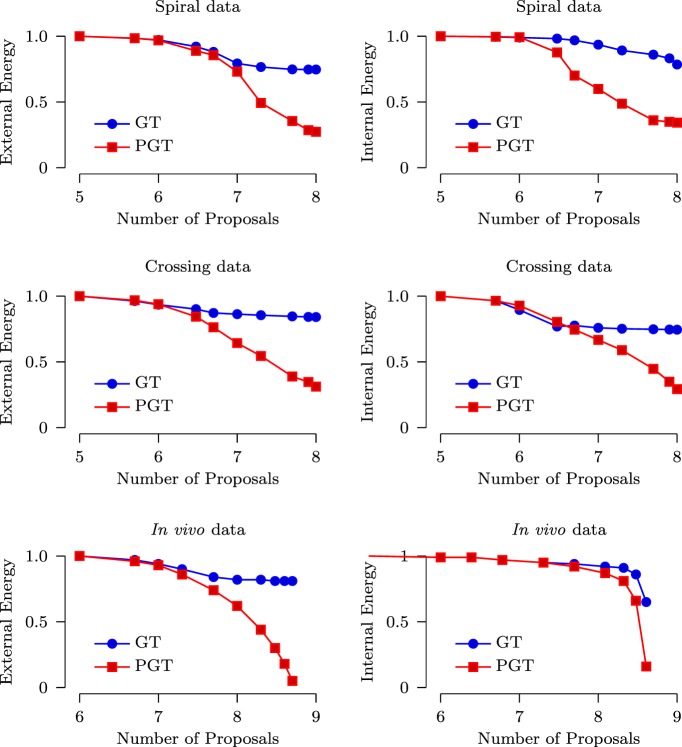
**Normalized external and internal energy plotted against the number of proposals (in logarithmic scale)**.

As mentioned in Section 2.1, the internal energy characterizes the smoothness of the fibers while the external energy characterizes the consistency between the predicted signal and the diffusion-weighted signal. We compared both the internal energy and external energy of GT and PGT (with 10 parallel threads). The parameters used for GT and PGT are set as recommended in Reisert et al. (2011). Figure 6 shows the results with respect to the number of generated proposals for the spiral, the crossing and the *in vivo* data. It can be observed that PGT outperforms GT with lower internal energy and external energy. It is interesting to note that when the energy curves of GT flatten out, those of PGT continue to decrease. This indicates that, when the adjustments of the fiber segments are done concurrently in multiple regions, a configuration with lower energy can be reached with greater ease.

#### 3.2.3 Computational Costs

**Figure 7:**
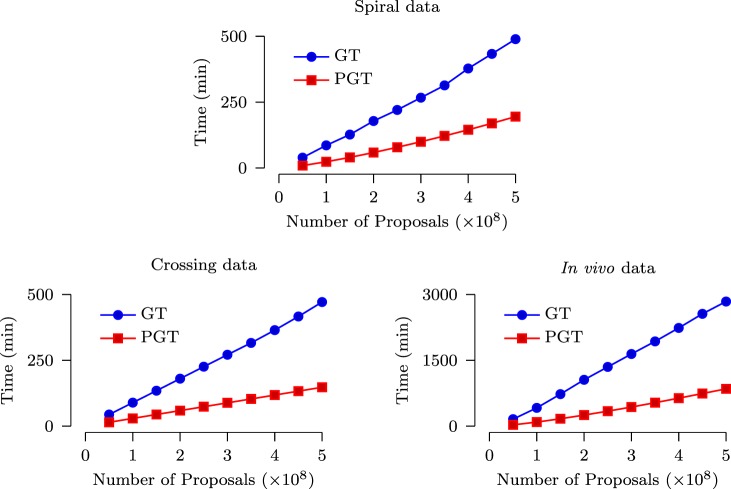
**Time costs of GT and PGT**.

The speed improvement given by PGT over GT was evaluated based on a computing cluster with 2.9 GHz Intel Xeon CPUs and 48 GB RAM. Figure 7 shows that, for both synthetic and *in vivo* data, PGT requires only less than approximately 1/3 of the time required by GT. Note that it is not possible to achieve the ideal 10× speed increase because the GT algorithm is only partially parallelized, as discussed in Section 2. Moreover, part of the time cost is associated with the computational overhead involved in the parallelized implementation.

#### 3.2.4 Tractography Results

**Figure 8:**
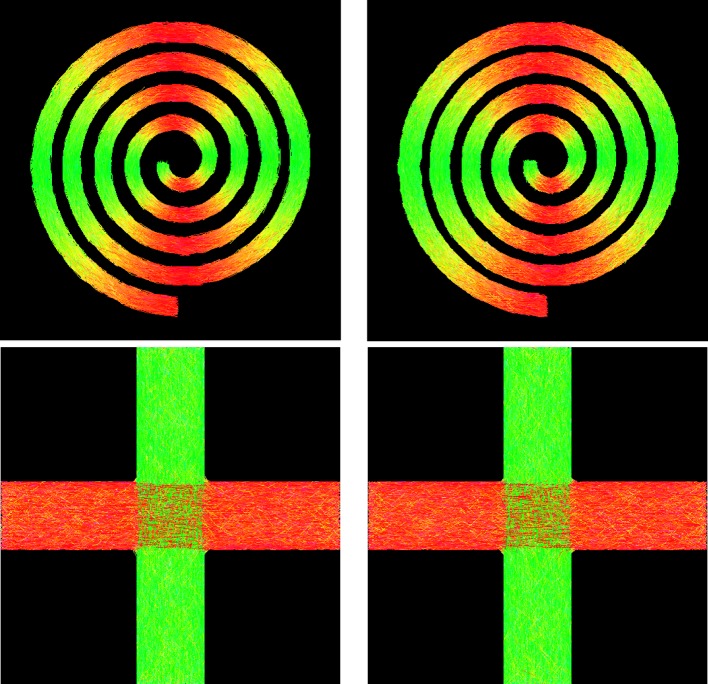
**Tractography results for synthetic data using (left) GT and (right) PGT**.

Figure 8 shows the tractography results of GT and PGT for the synthetic data. Both GT and PGT create reasonable and consistent fiber tracts that are in agreement with the data. For numerical evaluation, we computed the distance of fiber bundles given by GT and PGT using the distance defined in Yap et al. (2011a). The distance between two fiber bundles F and G is defined as

(13) $$\frac{1}{\left|F|+|G\right|}\left[\underset{F_{i}\in F}{\sum }\;\underset{G_{j}\in G}{\min}\;d\left(F_{i},G_{j}\right)+\underset{G_{i}\in G}{\sum }\;\underset{F_{j}\in F}{\min}\;d\left(G_{i},F_{j}\right)\right]$$

where *d* (*F_i_*, *G_j_*) is a pairwise distance between fibers *F_i_* ∈ F and *G_i_* ∈ G, which in our case, is defined as the mean of the closest distances calculated for all points on fiber *F_i_* to fiber *G_i_*. When two fiber tracts are identical, the value returned is zero. The respective distances for the spiral and the cross are 1.46 mm and 0.52 mm, indicating high similarity between the tractography results of GT and PGT. Despite the visual similarity between the GT and PGT, PGT yields in general a higher number of fiber tracts with greater lengths. Therefore, some dissimilarities exist between the results given by the two algorithms.

**Figure 9:**
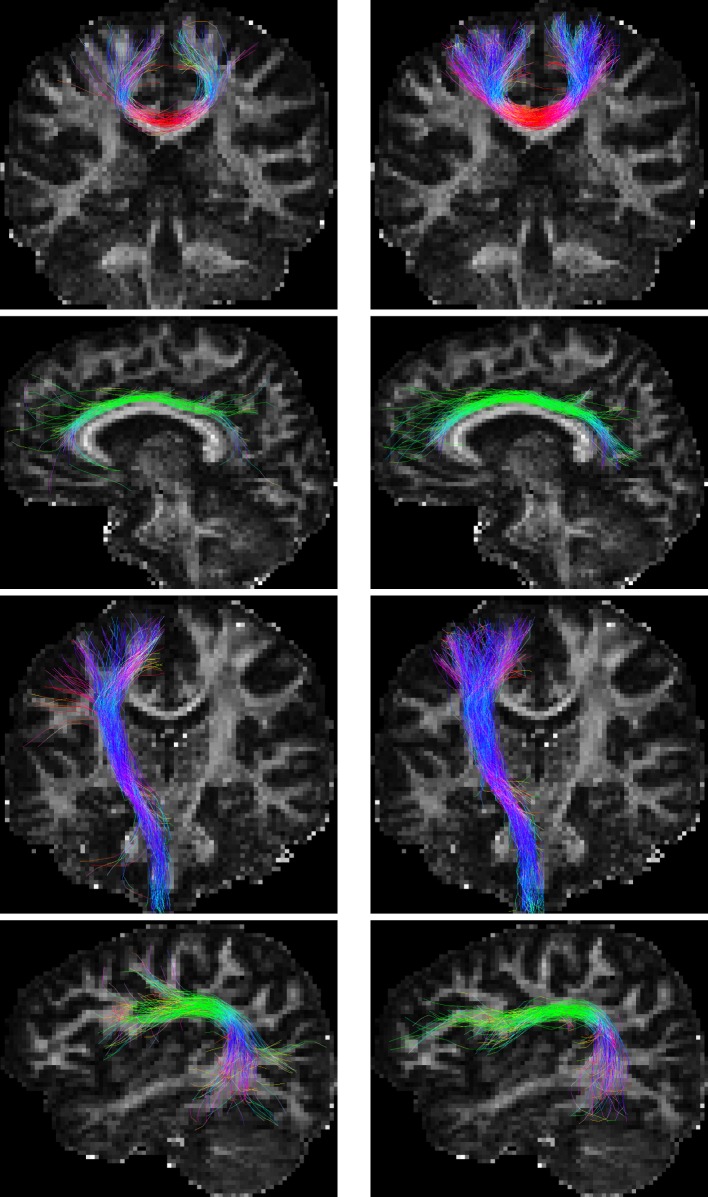
**Fiber bundles given by (left) GT and (right) PGT**. From top to bottom are the CC-PRC, CGC, CST, and ARC tracts, respectively.

For the *in vivo* data, fiber bundles extracted with multiple ROIs (Wakana et al., 2007) are shown in Figure 9. The extracted fiber tracts include (Jin et al., 2013, 2014): (1) association tracts such as the cingulum tract (CGC); (2) the arcuate fasciculus (ARC), a part of the superior longitudinal fasciculus; (3) projection tracts such as the corticospinal tract (CST); and (4) commissural tracts such as a segment of corpus callosum projecting to both precentral gyri (CC-PRC). PGT results in fiber bundles that are similar to, or even better than, GT, but in a fraction of time. The distances between the fiber tracts generated by GT and PGT are as follows: CGC: 3.78 mm; ARC: 4.86 mm; CST: 3.63 mm; and CC-PRC: 5.23 mm. Note that the fiber tracts generated by the two algorithms are not totally similar. PGT in general generates more tracts with greater lengths.

#### 3.2.5 Connectivity

**Figure 10:**
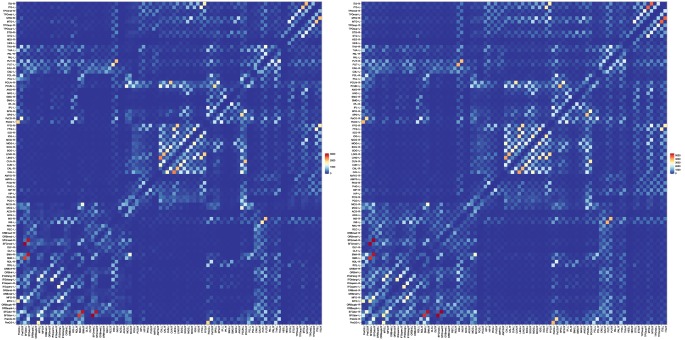
**Fiber count based connectivity network given by (left) GT and (right) PGT**.

Features derived from structural connectivity networks provide rich information for identifying brain disorders due to its comprehensive characterization of connections between different brain regions (Wee et al., 2012). The Automated Anatomical Labeling (AAL) atlas used the main sulci as the landmarks to parcellate a single adult brain data into 90 ROIs (Tzourio-Mazoyer et al., 2002). We mapped the atlas to the *in vivo* data using a deformable registration algorithm (Avants et al., 2011) and computed the number of fibers connecting each pair of ROIs. The results, shown in Figure 10, indicate that the PGT yields a connectivity map that is consistent with GT, albeit with more fibers. It can also be observed that PGT is also able to detect/strengthen weak connections that are missed by GT.

## 4 Discussion

The performance of PGT is dependent on the parameter *K*. A bigger *K* results in a greater degree of parallelization and tractography can be completed within a less amount of time. However, *K* is limited by the requirement of statistical independence of the configurations of the fiber segments in the *K* subregions. That is, in practice, it is not possible to infinitely partition a finite region. Moreover, a large K also implies that the configuration in each subregion will converge with a less number of iterations, requiring more frequent re-partitioning. This increases the computation overhead and decreases efficiency.

To further increase parallelization, we can allow overlapping of subregions by wrapping data access in a mutex. The mutex will provide a lock–unlock mechanism for mutually exclusive updating of these subregions to avoid *data race*, where two or more threads access the same memory location concurrently. Overlapping of subregions will allow more threads to be used to speed up PGT.

One can also partition the brain regions using structurally adaptive subregions that fit better to the white matter. This will allow more irregularly shaped subregions to be fitted in a region of limited size. This will hence allow us to spawn more threads and hence increase parallelism.

## 5 Conclusion

The proposed algorithm helps reduce the time cost associated with the global optimization process required in global tractography. We run in parallel multiple independent chains of MCMC on a number of subregions, resulting in faster convergence and producing results that are comparable to the non-parallelized version. Future implementation based on GPUs will further improve the speed of global tractography and hence its feasibility in large-scale studies.

## Author Contributions

Conceived and designed the experiments: HW and P-TY. Performed the experiments: HW and GC. Analyzed the data: HW, GC, P-TY, and YJ. Contributed reagents/materials/analysis tools: HW, P-TY, GC. Wrote the paper: HW, YJ, P-TY, and DS.

## Conflict of Interest Statement

The authors declare that the research was conducted in the absence of any commercial or financial relationships that could be construed as a potential conflict of interest.
